# Gsα Controls Cortical Bone Quality by Regulating Osteoclast Differentiation via cAMP/PKA and β-Catenin Pathways

**DOI:** 10.1038/srep45140

**Published:** 2017-03-24

**Authors:** Girish Ramaswamy, Hyunsoo Kim, Deyu Zhang, Vitali Lounev, Joy Y. Wu, Yongwon Choi, Frederick S. Kaplan, Robert J. Pignolo, Eileen M. Shore

**Affiliations:** 1 Department of Orthopaedic Surgery, Perelman School of Medicine at the University of Pennsylvania, Philadelphia, PA 19104, USA; 2 Center for Research in FOP and Related Disorders, Perelman School of Medicine at the University of Pennsylvania, Philadelphia, PA 19104, USA; 3 Department of Pathology and Laboratory Medicine, Perelman School of Medicine at the University of Pennsylvania, Philadelphia, PA 19104, USA; 4 Division of Endocrinology, Stanford University School of Medicine, Stanford, CA 94305, USA; 5 Department of Medicine, Perelman School of Medicine at the University of Pennsylvania, Philadelphia, PA 19104, USA; 6 Division of Geriatric Medicine and Gerontology, Mayo Clinic College of Medicine, Rochester, MN 55905, USA; 7 Department of Genetics, Perelman School of Medicine at the University of Pennsylvania, Philadelphia, PA 19104, USA

## Abstract

Skeletal bone formation and maintenance requires coordinate functions of several cell types, including bone forming osteoblasts and bone resorbing osteoclasts. Gsα, the stimulatory subunit of heterotrimeric G proteins, activates downstream signaling through cAMP and plays important roles in skeletal development by regulating osteoblast differentiation. Here, we demonstrate that Gsα signaling also regulates osteoclast differentiation during bone modeling and remodeling. *Gnas*, the gene encoding Gsα, is imprinted. Mice with paternal allele deletion of *Gnas (Gnas*^+/p−^) have defects in cortical bone quality and strength during early development (bone modeling) that persist during adult bone remodeling. Reduced bone quality in *Gnas*^+/p−^ mice was associated with increased endosteal osteoclast numbers, with no significant effects on osteoblast number and function. Osteoclast differentiation and resorption activity was enhanced in *Gnas*^+/p−^ cells. During differentiation, *Gnas*^+/p−^ cells showed diminished pCREB, β-catenin and cyclin D1, and enhanced Nfatc1 levels, conditions favoring osteoclastogenesis. Forskolin treatment increased pCREB and rescued osteoclast differentiation in *Gnas*^+/p−^ by reducing Nfatc1 levels. Cortical bone of *Gnas*^+/p−^ mice showed elevated expression of Wnt inhibitors sclerostin and Sfrp4 consistent with reduced Wnt/β-catenin signaling. Our data identify a new role for Gsα signaling in maintaining bone quality by regulating osteoclast differentiation and function through cAMP/PKA and Wnt/β-catenin pathways.

Embryonic and early postnatal skeletal growth predominantly occurs through bone modeling, with bone resorption and formation occurring independently of each other to control bone size and shape. After skeletal development is complete, bone remodeling occurs throughout life with resorption and formation balanced at the bone surfaces in order to repair and maintain skeletal homeostasis^1^,^2^,^3^. However, during inflammation, diseases such as osteoporosis, and cancer metastasis, the balance between bone formation by osteoblasts and resorption by osteoclasts is shifted to increased resorption leading to reduced or poor quality bone^4^,^5^. Therefore, it is imperative to understand how osteoclast differentiation and function are regulated in order to improve therapeutic approaches.

Bone modeling and remodeling are precisely coordinated through interactions and regulation by multiple genes and signaling pathways including *GNAS*, which encodes the α-subunit of stimulatory G-protein (Gsα) of adenylyl cyclase and activates cAMP signaling. Gsα deletion in osteogenic lineage cells expressing osterix and col1a has demonstrated that Gsα plays important roles during skeletal development by regulating mesenchymal cell commitment, osteoblast differentiation, and mineralization without affecting osteoclast function^6^,^7^,^8^,^9^. Recent studies have also suggested that cAMP levels are regulated by several mechanisms including Wnt signaling, adenylyl cyclase, and Ca^2+^/Calmodulin-dependent kinases to delicately control osteoclastogenesis^10^,^11^. In addition, in fibrous dysplasia, a clinical condition in which activating Gsα mutations cause elevated cAMP levels, increased osteoclasts occur primarily in response to osteoblast secretion of interleukin-6^12^,^13^. There has been no evidence of a direct role for Gsα in osteoclast differentiation and function, and the impact of Gsα on skeletal bone quality during modeling and remodeling remains insufficiently understood.

The *GNAS* gene is a complex locus that expresses multiple transcripts^14^,^15^,^16^. The *GNAS* locus is imprinted, expressing some transcripts specifically from the paternally-inherited allele and others from the maternally-inherited allele. Gsα mRNA is bi-allelically expressed in most cells^17^. These different allele-specific expression patterns are reflected in human diseases and mouse models that are phenotypically distinct depending on the allele carrying a *GNAS* inactivating mutation^18^,^19^. Mice with paternally-inherited heterozygous deletion of either Gsα exon 1 or exon 2 have shorter body lengths and lower body weights, while mice with maternal inheritance of the deletion of either of the exons are short but obese^20^. These data suggested that heterozygous *GNAS* inactivation impacts skeletal bone by affecting growth plate development and bone formation. But how Gsα deletion affects skeletal bone quality during modeling and remodeling, and whether these effects differ with paternal and maternal inheritance of the mutation, has not been examined.

In this study, we investigated the effects of heterozygous *GNAS* inactivation in mice on skeletal bone during modeling and remodeling. We examined trabecular and cortical bone from mice with paternal or maternal inheritance of Gsα deletion, and the roles of Gsα signaling on osteoblast and osteoclast formation and function. Our data reveal that heterozygous *Gnas* inactivation negatively affects cortical bone quality, with mutation of the paternal allele causing more severe effects than maternal mutations. We further determined that paternally inherited Gsα deletion alters cortical bone maintenance not through effects on osteoblasts and bone formation, but through enhanced osteoclast differentiation and increased bone resorption, and that these effects are mediated by Gsα signaling via cAMP/PKA and Wnt/β-catenin pathways.

## Results

### Paternal inheritance of heterozygous *Gnas* inactivation adversely affects cortical bone quality during bone modeling and remodeling

**Figure 1:**
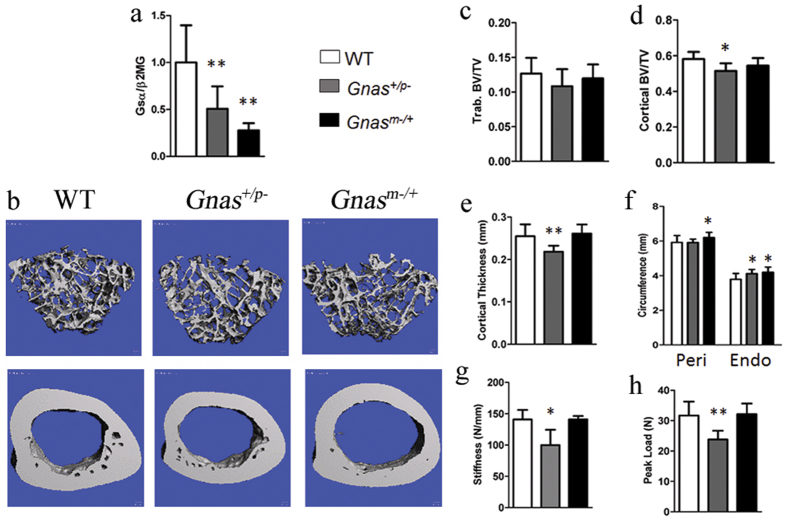
Paternal but not maternal heterozygous *Gnas* deletion causes reduction in cortical bone quality in 9 month old mice. (**a**) mRNA expression of Gsα in cortical bone was reduced in heterozygous *Gnas* mutants compared to WT by qRT-PCR. There was no statistical difference between *Gnas*^+/p−^ and *Gnas*^m−/+^. (**b**) Representative 3D μCT images of (top) trabecular and (bottom) cortical bone. (**c**) No differences in trabecular bone volume fraction were observed between the groups. Decreased (**d**) cortical bone volume fraction, (**e**) cortical thickness and (**g**) stiffness and (**h**) peak load in *Gnas*^+/p−^ mice but not *Gnas*^m−/+^ mice as compared to WT. (**f**) Endosteal circumference (Endo) measured at the femoral mid-shaft is increased with no change in periosteal circumference (Peri) in *Gnas*^+/p−^ mice while both are increased in *Gnas*^m−/+^ mice. Data represent mean ± SD. N = 13 WT, 7 *Gnas*^+/p−^ and 5 *Gnas*^m−/+^ animals. *p < 0.05, **p < 0.01.

To determine the role of Gsα signaling on skeletal bone quality, we used an established mouse model of heterozygous *Gnas* inactivation^20^. Since *Gnas* is an imprinted locus, we examined mice with inactivation of the paternally-inherited *Gnas* allele (*Gnas*^+/p−^) and the maternally-inherited *Gnas* allele (*Gnas*^m−/+^). qRT-PCR of mRNA from cortical bone confirmed significant reduction in Gsα expression in both *Gnas*^+/p−^ and *Gnas*^m−/+^ mice (Fig. 1a).

In most mouse strains, rapid accrual of bone occurs until 3 months of age, stabilizes by 6–8 months, and then is maintained by remodeling^21^. To evaluate the effects of heterozygous *Gnas* deletion on adult bone remodeling, we analyzed mice at 3 and 9 months of age. At both ages, *Gnas*^+/p−^ mice weighed less and were shorter (body and femur length were measured; femur length is reported in this study) than WT controls while *Gnas*^m−/+^ mice had increased body weight with no difference in femur length (Supplementary Table 1), consistent with reported results^20^. Previously, histomorphometry of distal femurs in 3-month-old animals had identified no differences vs. controls in trabecular bone volume fraction or osteoblast function in either *Gnas*^+/p−^ or *Gnas*^m−/+^ mice^20^. Trabecular microarchitecture was not investigated in detail and no analysis of cortical bone was performed^20^. To examine trabecular microarchitecture, distal metaphyseal femurs of 3- (Supplementary Table 1) and 9-month-old mice were examined by μCT (Fig. 1 and Supplementary Table 1). Overall, trabecular bone volume fraction (BV/TV) and microarchitecture (Fig. 1b,c and Supplementary Table 1) in distal femurs showed no significant differences at both ages in *Gnas*^+/p−^ and *Gnas*^m−/+^ mice compared to littermate controls, consistent with previous results^20^, however trends of marginal reduction, notably trabecular BV/TV and trabecular thickness, were observed in *Gnas*^+/p−^ mice. The trabecular bone of lumbar vertebrae (data not shown) also appeared unaffected in both paternally- and maternally-inherited *Gnas* mutants compared to controls.

Cortical bone μCT analyses of mid-diaphyses of adult 3- and 9-month-old *Gnas*^m−/+^ femurs revealed significantly increased total cortical bone volume as well as greater periosteal and endosteal circumference, consistent with the increased body weight of these mutants (Fig. 1f and Supplementary Table 1) relative to WT. No differences in cortical thickness or cortical bone volume fraction were observed (Fig. 1b,d,e). In contrast, *Gnas*^+/p−^ mice showed significant reductions in total cortical bone volume, cortical thickness, and cortical bone volume fraction at both 3 (data not shown) and 9 months (Fig. 1b,d,e and Supplementary Table 1). A larger endosteal circumference with no change in periosteal circumference suggested an increase in endosteal resorption in *Gnas*^+/p−^ mice compared to WT (Fig. 1f). Cortical porosity was marginally increased in the *Gnas*^+/p−^ mice at both ages, but not statistically significant (Supplementary Table 1). *Gnas*^+/p−^ femurs at both ages were weaker by three-point bending tests, with significant reductions in peak load and stiffness compared to WT; bone strength was unaffected in *Gnas*^m−/+^ mice at either age (Fig. 1g,h and Supplementary Table 1). These results indicated that paternal-allele inactivation of *Gnas* affects cortical bone quality during stages of adult bone remodeling.

**Figure 2:**
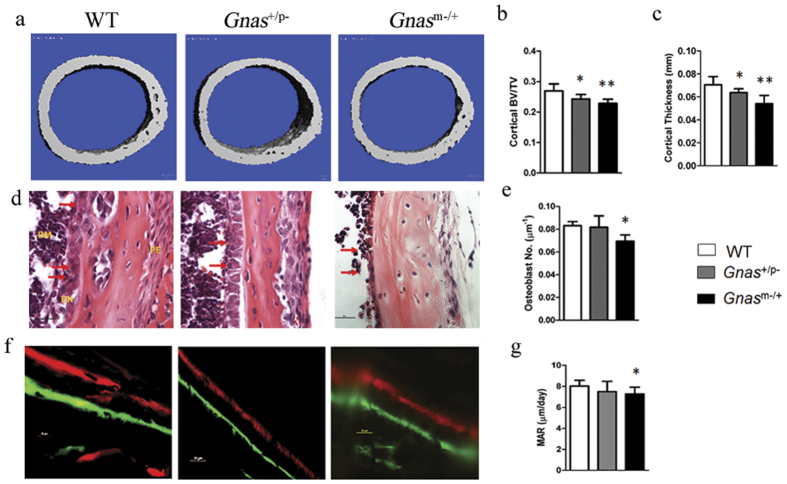
Cortical bone defects in *Gnas*^+/p−^ mice are not due to defects in osteoblast numbers and function in *Gnas*^+/p−^ mice. (**a**) Representative 3D μCT images of cortical bone from mice at 2 weeks of age. (**b**) Cortical bone volume fraction and (**c**) cortical thickness measurements by μCT scans at the mid-diaphyseal region of femurs were significantly reduced in both *Gnas*^+/p−^ and *Gnas*^m−/+^ 2-week-old mice. (**d**) H&E staining of cortical bone shows osteoblasts (arrows) lining the endocortical surface. (**f**) Double labeling of cortical bone surface with calcein and xylenol orange in 2-week-old mice. Quantification of osteoblast number (**e**) and mineral apposition rate (**g**) along the endocortical surface showed reduction in *Gnas*^m−/+^ but not *Gnas*^+/p−^ when compared to WT mice. Data represent mean ± SD. N = 7–11 animals per group for μCT and 5–6 animals per group for histology. *p < 0.05, **p < 0.01.

To determine effects of *Gnas* inactivation on early skeletal development and bone modeling, we examined cortical bone in 2-week-old mice by μCT and mechanical testing. As in older mice, no statistically significant differences in trabecular bone were found although trends of reduced BV/TV and trabecular thickness were observed in *Gnas*^+/p−^ mice. However, in contrast to older mice (Fig. 1), both *Gnas*^+/p−^ and *Gnas*^m−/+^ young mice showed cortical bone defects, with significant decreases in cortical thickness and bone volume fraction (Fig. 2a–c, Supplementary Table 1). At this age, both *Gnas*^+/p−^ and *Gnas*^m−/+^ mice were lower in body weight and femur length compared to WT, with paternally inherited mutants showing greater reduction than the maternal allele mutants. Using three-point bending tests, femurs from both mutants showed significant reductions in stiffness and peak load (Supplementary Table 1) consistent with the μCT data.

Collectively, these results show that although deletion of either parental *Gnas* allele affects cortical bone during modeling, only inactivation of the paternally-inherited allele impacts cortical bone quality during remodeling.

### Osteoblast numbers and function are unaffected in *Gnas* ^+/p−^ mice

Several studies demonstrated that ablation of Gsα signaling causes skeletal defects, including lower trabecular and cortical bone volume, due to effects on osteoblasts and bone formation^6^,^8^. These data were mainly observed in mice with Gsα deletion specifically in cells of the osteoblast lineage.

To investigate skeletal bone effects of heterozygous *Gnas* inactivation in osteoblasts, we used conditional *Gnas* heterozygous mice with paternal or maternal allele Gsα deletion and Cre expression driven by the early osteoblast marker osterix (Osx-Cre; *Gnas*^+/p−^ and Osx-Cre; *Gnas*^m−/+^). Unlike previous studies in *Gnas* null osteoblasts, μCT analyses revealed no differences at 6 weeks of age in either trabecular or cortical bone (Supplementary Figure 1a,b) in these mutants compared to control mice (*Gnas*^+/+^, *Gnas*^*fl*/+^ and Osx-Cre; *Gnas*^+/+^).

To determine potential contributions of osteoblast numbers and function to our observed cortical bone defects in 2-week-old mutant mice, we quantified osteoblasts and mineral apposition rates (MAR). While both parameters in *Gnas*^+/p−^ mice were similar to WT controls, *Gnas*^m−/+^ mice showed a marginal but statistically significant decrease in both osteoblast number and MAR compared to WT controls (Fig. 2d–g). This suggested that paternally and maternally inherited Gsα mutants affect cortical bone during development differently and support that cortical bone defects in *Gnas*^+/p−^ mice were not due to effects in osteoblasts but possibly to effects on other cell types.

### Gnas^+/p−^ mice show increased endosteal osteoclasts during remodeling

**Figure 3:**
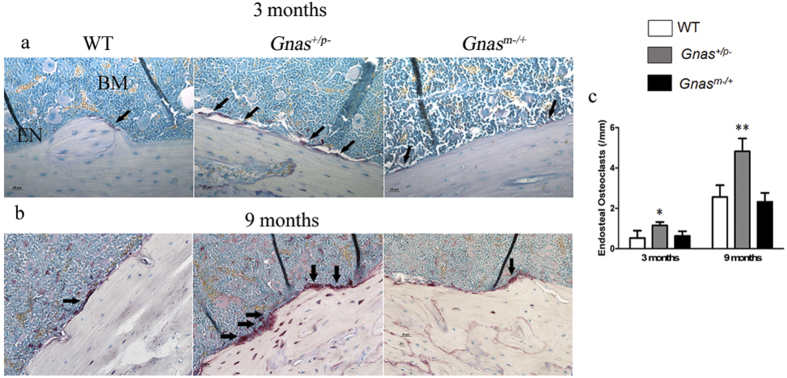
Mice with paternal inheritance of heterozygous deletion of *Gnas* have elevated numbers of endosteal osteoclasts. At (**a**) 3 months and (**b**) 9 months of age, *Gnas*^+/p−^ exhibit increased endocortical osteoclasts (arrows) detected by TRAP staining compared to WT and *Gnas*^m−/+^ mice. (**c**) Quantification of endocortical osteoclast number at the diaphyseal region in WT, *Gnas*^+/p−^ and *Gnas*^m−/+^ mice at 3 and 9 months of age. Data represent mean ± SD. N = 4–8 animals per group. *p < 0.05. EN – endosteum, BM – bone marrow.

Cortical bone remodeling is a balance between osteoblast and osteoclast activity. Since osteoblast numbers and function were unaffected in paternal *Gnas* mutants, we hypothesized that cortical bone defects in these mice could be due to altered osteoclasts. To determine osteoclast numbers, femurs from WT and *Gnas*^+/p−^ and *Gnas*^m−/+^ mutants at 2 weeks, and 3 and 9 months of age were stained with TRAP and numbers of TRAP positive multi-nucleated osteoclasts along the cortical surface were quantified. At 2 weeks of age, when bone modeling is occurring, TRAP staining was primarily observed on the periosteal surface, and no differences among the genotypes were detected (data not shown). At 3 months of age, the numbers of TRAP positive osteoclasts were significantly elevated in *Gnas*^+/p−^ mice (Fig. 3a,c) while *Gnas*^m−/+^ were similar to WT. At 9 months of age (Fig. 3b), the numbers of TRAP positive osteoclasts observed in all genotypes along the endosteal surface were higher than at 3 months. *Gnas*^m−/+^ femurs showed no differences in numbers of TRAP positive multi-nucleated osteoclasts compared to WT as was found at 3 months, while *Gnas*^+/p−^ mice showed a dramatic increase in endosteal osteoclast numbers (Fig. 3c), consistent with the cortical bone defects detected in *Gnas*^+/p−^ mice. No differences among the genotypes were observed for osteoclast numbers on trabecular bone surfaces (Supplementary Figure 2). These data suggest that inactivation of the paternally-inherited allele of *Gnas* increases osteoclast numbers and enhances endosteal resorption resulting in cortical bone loss.

### Paternal allele Gnas inactivation enhances osteoclast differentiation and resorption *in vitro*

Since only *Gnas*^+/p−^ but not *Gnas*^m−/+^ mice showed persistent defects in cortical bone quality concomitant with increase in endosteal osteoclasts, we investigated whether paternal allele *Gnas* inactivation has a direct effect on either the osteoclast progenitor population or osteoclast differentiation and function. In order to study the progenitor population, osteoclast precursors were counted from the bone marrow of WT and *Gnas*^+/p−^ mice at 7–9 weeks of age by flow cytometry^22^. The numbers of osteoclast precursor cells, namely the CD3^−^ CD45R^−^ CD11b^−/low^ CD115^high^ population, were the same in mutant and WT bone marrow (Supplementary Figure 3) indicating that cortical bone defects in *Gnas*^+/p−^ mice are not caused by increased osteoclast precursors in the bone marrow.

**Figure 4:**
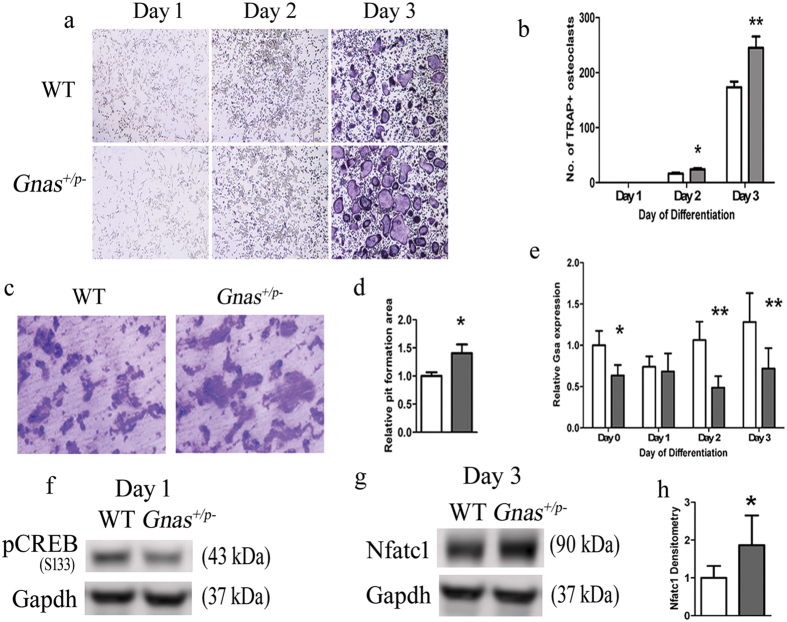
Paternal inheritance of *Gnas* inactivation enhances osteoclast differentiation and resorption activity of osteoclasts. (**a**) Differentiation of bone marrow macrophages (BMMs) from 7–9 week old WT and *Gnas*^+/p−^ mice into osteoclasts. (**b**) Quantitation of TRAP^+^ multi-nucleated cells (≥3 nuclei) at days 1–3 of differentiation shows increased *Gnas*^+/p−^ osteoclasts at days 2 and 3 of differentiation. (**c**) Osteoclasts differentiated from BMMs were seeded on bone slices for 48 h and (**d**) the relative resorption area measured; resorption activity was greater with *Gnas*^+/p−^ osteoclasts compared to WT. (**e**) mRNA expression of Gsα was reduced over time in *Gnas*^+/p−^ cells during osteoclast differentiation. β2-microglobulin was used for normalization and WT values were set to 1. (**f**) pCREB was lower during osteoclast differentiation in *Gnas*^+/p−^ cells as compared to WT cells. (**g,h**) Nfatc1 from whole cell lysate was significantly elevated in *Gnas*^+/p−^ cells as compared to WT cells at day 3 of osteoclast differentiation. Data represent mean ± SD. Experiments were performed at least 3 times with n = 2–3 animals per group per experiment. Total of 5–7 animals per group from 3 experiments used for quantification. For osteoclast differentiation and pit formation, cells were seeded in triplicates in 96-well plate and on bone slices respectively. For pit formation, resorption area mean of WT was set to 1. *p < 0.05; **p < 0.01.

Upon differentiation to osteoclasts *in vitro*, bone marrow macrophages (BMM) from *Gnas*^+/p−^ produced significantly more TRAP positive multi-nucleated (≥3 nuclei) osteoclasts at 2 and 3 days of differentiation compared to WT (Fig. 4a and b). qRT-PCR confirmed that Gsα expression was significantly decreased in the cells from *Gnas*^+/p−^ during osteoclast differentiation relative to WT (Fig. 4e). cAMP levels showed trends of reduction in *Gnas*^+/p−^ cells relative to WT (Supplementary Figure 4f). In addition, osteoclasts from *Gnas*^+/p−^ mice on dentine slices formed larger resorption pits compared to WT osteoclasts (Fig. 4c,d). When BMMs from *Gnas*^m−/+^ were cultured, they produced more mature osteoclasts that formed larger resorption pits than WT littermate controls (Supplementary Figure 5a,b), however these differences in *Gnas*^m−/+^ cells were less than in *Gnas*^+/p−^ cells, with a 40% increase in osteoclast differentiation and resorption in *Gnas*^+/p−^ relative to controls vs. only 15% increase in these parameters in *Gnas*^m−/+^. These results support that reduced Gsα signaling enhances osteoclast differentiation and osteoclast resorption activity, with greater effects from deletion of the paternal *Gnas* allele than the maternal allele.

### Decreased cAMP/PKA pathway, increased Nfatc1 and enhanced osteoclast differentiation in Gnas^+/p−^ mice

Gsα signals primarily through adenylyl cyclase and the cAMP/PKA pathway^23^,^24^,^25^. Recent studies implicated this pathway as inhibitory to osteoclast differentiation by phosphorylating Nfatc1, an important transcription factor for osteoclast differentiation^11^,^26^. Adenylyl cyclase activity is decreased in *Gnas*^+/p−^ mice^20^ raising the possibility that decreased cAMP leads to decreased phospho-Nfatc1 and increased osteoclasts. To investigate this mechanism, we analyzed protein levels of pCREB, a downstream activator of the cAMP/PKA pathway during osteoclast differentiation. We found that pCREB protein levels were marginally reduced at day 0 and significantly reduced at day 1 of osteoclast differentiation in cells from *Gnas*^+/p−^ mice (Fig. 4f and Supplementary Figures 4a,b). We investigated whether this decreased cAMP signaling is associated with altered Nfatc1 levels and determined that both total and nuclear Nfatc1 protein was highly up-regulated at day 3 of osteoclast differentiation in *Gnas*^+/p−^ osteoclasts compared to WT (Fig. 4g,h and Supplementary Figures 4d,e) suggesting that paternal *Gnas* allele deletion reduces pCREB during early differentiation and enhances Nfatc1 at a protein level to impact osteoclastogenesis.

**Figure 5:**
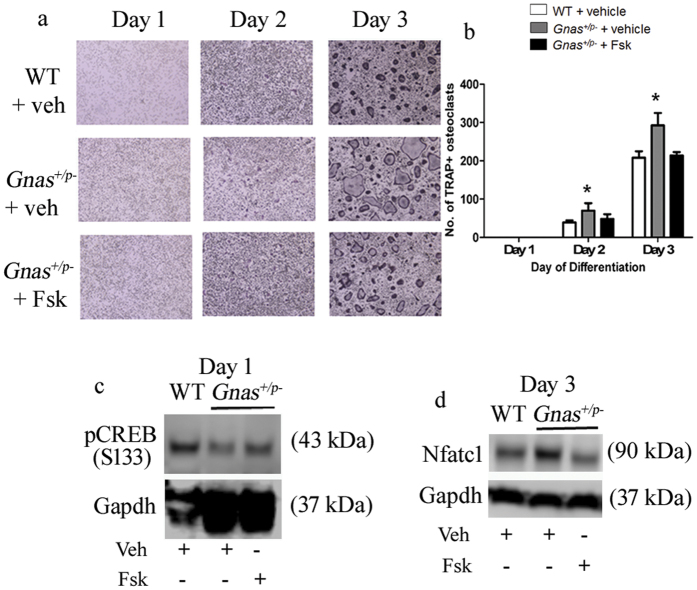
Forskolin (Fsk) rescues the osteoclast differentiation phenotype of cells from *Gnas*^+/p−^ mice. (**a**,**b**) Forskolin treatment during osteoclast differentiation rescued the increased osteoclast numbers in *Gnas*^+/p−^ mice. (**c,d)** Forskolin treatment of *Gnas*^+/p−^ cells increased pCREB (**c**) and reduced Nfatc1 (**d**) to levels comparable to WT. Experiments were performed at least 3 times with n = 1–2 animals per group per experiment. Total of 5 animals per group used for quantification. *p < 0.05.

Forskolin inhibits Nfatc1-induced osteoclastogenesis by elevating adenylyl cyclase and PKA activity^11^. We therefore hypothesized that treatment with forskolin would rescue the increased osteoclast differentiation by *Gnas*^+/p−^ cells. In order to test this, we induced osteoclast differentiation of WT and *Gnas*^+/p−^ osteoclast precursor cells in the presence or absence of forskolin. Treatment of *Gnas*^+/p−^ cells with forskolin abolished the increase in TRAP^+^ multi-nucleated osteoclasts to levels comparable to WT (Fig. 5a,b). In addition, forskolin increased pCREB (Fig. 5c) and decreased Nfatc1 (Fig. 5d) to levels comparable to WT. Together, these results demonstrate that activation of PKA rescues pCREB, and regulates Nfatc1 levels and osteoclast differentiation in *Gnas*^+/p−^ cells.

### β-catenin and its target cyclin D1 are reduced in Gnas^+/p−^ mice

**Figure 6:**
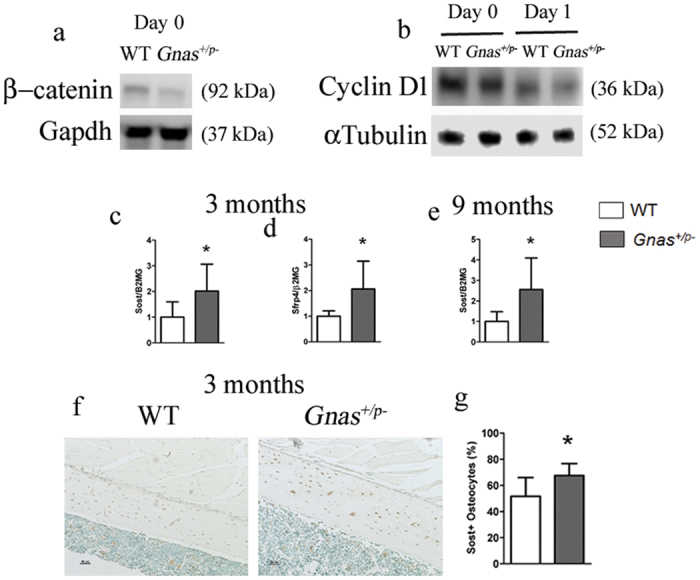
Wnt/β-catenin-mediated cyclin D1 signaling is downregulated in *Gnas*^+/p−^ mice. Western blots of (**a**) β-catenin and (**b**) cyclin D1 at days 0 and 1 during osteoclast differentiation showed reduced levels in *Gnas*^+/p−^ cells during osteoclast differentiation. mRNA expression of the Wnt pathway inhibitors Sost (**c**) and Sfrp4 (**d**) at 3 months and Sost at 9 months (**e**) is increased in cortical bone from *Gnas*^+/p−^ mice as compared to WT by qRT-PCR. β2-microglobulin was used for normalization and WT values were set to 1. (**f**) Immunohistochemistry of sclerostin in the mid-diaphyseal region of femurs from 3-month old WT and *Gnas*^+/p−^mice. (**g**) Quantitation of IHC detection shows increase in Sclerostin positive osteocytes in *Gnas*^+/p−^ cortical bone as compared to WT. Data represent mean ± SD. Western blots were performed twice with n = 2–3 animals per group per experiment. N = 5–8 animals per group for real time PCR. N = 6 animals per group for IHC. *p <** **0.05.

Wnt signaling inhibits osteoclast differentiation through canonical β-catenin-dependent^27^ and non-canonical^10^ pathways. β-catenin and its transcriptional target cyclin D1 have been shown to play biphasic roles during osteoclast proliferation and differentiation^28^. Their expression is required for osteoclast precursor proliferation, and both are activated by M-CSF but suppressed by RANKL during osteoclast differentiation. Since mice with Gsα deletion in osteoblasts^6^ and osteocytes^29^ exhibit decreased Wnt/β-catenin signaling, we examined whether reduction in Wnt/β-catenin signaling in *Gnas*^+/p−^ mice could lead to increased osteoclast differentiation. To determine the levels of Wnt/β-catenin signaling, total β-catenin from cell lysates during osteoclast differentiation were detected by immunoblot. Under normal circumstances, RANKL suppresses β-catenin and cyclin D1 during osteoclast differentiation. Indeed, we found that both WT and *Gnas*^+/p−^ cells showed reduction in β-catenin (data not shown) and cyclin D1 (Fig. 6b) in response to RANKL. However, comparison of WT and *Gnas*^+/p−^ cells showed that β-catenin and cyclin D1 were more greatly decreased in *Gnas*^+/p−^ cells than WT (Fig. 6a,b), supporting that inappropriately low levels of β-catenin and its downstream cyclin D1 enhance osteoclast differentiation in *Gnas*^+/p−^ mice.

To determine whether Wnt/β-catenin signaling is altered in skeletal bone cells of *Gnas*^+/p−^ mice, expression of Wnt targets and inhibitors from the diaphyses of femurs and tibiae from 3- and 9- month-old mice was quantified. While there was no difference in expression of the Wnt target gene Lef1 (data not shown), mRNA levels of the Wnt inhibitors Sost and Sfrp4 were up-regulated in both 3 and 9-month-old *Gnas*^+/p−^ mice (Fig. 6c–e). A higher percent of osteocytes positive for Sclerostin in femurs of 3-month-old *Gnas*^+/p−^ mice was detected by immunohistochemistry (Fig. 6f,g). Collectively, these data support that an overall increase in Wnt inhibitors causes a decrease in β-catenin/cyclin D1 leading to increased osteoclastogenesis in paternally-inherited *Gnas* inactivation mutants.

## Discussion

Human diseases caused by *GNAS* mutations provide insight into the roles of *GNAS* in bone formation and have identified regulatory functions in osteoblasts. In progressive osseous heteroplasia (POH) and pseudohypoparathyroidisms 1A (PHP1A), heterozygous *GNAS/Gnas* inactivation and decreased Gsα signaling cause heterotopic ossification (extra-skeletal bone formation) in soft tissues such as subcutaneous fat and muscle^15^,^24^,^30^,^31^. Fibrous dysplasia (FD), caused by somatic activating mutations in *GNAS*, is characterized by woven bone lesions in skeletal bone due to defective osteoblast differentiation^32^,^33^,^34^; FD also shows increased osteoclastic bone resorption at the site of these lesions that is not a direct effect of Gsα signaling in osteoclasts but is a response by osteoclasts to increased interleukin-6 secretion by osteoblasts^12^.

In this study, we investigated the effects of heterozygous *GNAS* inactivation on skeletal bone during modeling and remodeling and unexpectedly identified a novel requirement for Gsα signaling to maintain bone quality through regulation of osteoclast differentiation via cAMP/PKA/pCREB and Wnt/β-catenin signaling pathways. Although we cannot exclude effects of the mutation on trabecular bone, we identified a stronger effect in cortical bone vs. trabecular bone and therefore focused our investigations on the mechanisms through which *Gnas*^+/p−^ causes decreased cortical bone quality. Cortical bone forms 80% of our skeletal mass and 80% of fractures occur at cortical bone sites^35^,^36^, therefore, it is clinically relevant to understand the signaling mechanisms that regulate cortical bone maintenance during normal remodeling as well as aging and disease.

The *GNAS* gene locus encodes several transcripts, with expression patterns dependent on the parent of origin of each allele^15^,^16^. In mice and humans, inactivation or deletion of the paternally vs. maternally-inherited *GNAS* allele results in different phenotypes. For example, PHP1A is caused by mutation of the maternally-inherited *GNAS* allele and leads to subcutaneous heterotopic ossification and hormone resistance. In contrast, POH which is mainly associated with paternally-inherited allele mutation, presents with more severe progression of heterotopic ossification into deep connective tissues such as the muscle and without hormone resistance^14^,^18^,^37^,^38^. The underlying mechanisms for these phenotypic differences are incompletely understood, and may be influenced by genomic and tissue-specific imprinting at the *GNAS* locus and/or differences in *GNAS* transcript expression.

In our study, heterozygous *Gnas* knockout mice with paternal (*Gnas*^+/p−^) vs. maternal (*Gnas*^m−/+^) inheritance of a *Gnas* deletion showed similar defects in cortical bone quality and strength during early development (bone modeling). This reduction in bone quality persisted during adult bone remodeling in *Gnas*^+/p−^ mice, however *Gnas*^m−/+^ mice recovered, showing bone qualities similar to *Gnas*^+/+^ control mice at adult bone remodeling stages.

Gsα signaling activates cAMP which phosphorylates PKA and CREB^24^,^39^. Previous studies of homozygous deletion of Gsα in osterix-expressing osteoblast precursors revealed adverse effects on both trabecular and cortical bone due to defective mesenchymal progenitor commitment to osteoblasts and terminal osteoblast differentiation^6^,^9^. Gsα deletion in col1α1-expressing osteoblasts showed decreased TRAP positive osteoclasts at the endocortical bone surface leading to an increase in cortical thickness; however, this analyses was limited to the bone modeling stage in newborn mice^9^. Furthermore, activation of cAMP and phosphorylation of PKA and CREB in osteoblasts was found to activate osteoclasts by increasing RANKL and inhibiting OPG (a decoy receptor for RANKL that impedes osteoclastogenesis)^40^,^41^. None of these studies examined the effects of Gsα signaling on osteoclast differentiation and function or its impact on postnatal bone quality during bone remodeling.

Given this previous evidence that Gsα affects osteoblasts and bone formation, we examined whether the reduced skeletal bone quality in *Gnas* mutant mice was due to impaired osteoblast numbers or function. We found no significant effects on osteoblast number and function in the paternal mutants while maternal mutants unexpectedly showed a marginal reduction in both parameters. Although statistically significant, these effects in maternal *Gnas* mutants are small and insufficient to draw conclusions about functional effects. Future studies to further investigate proliferation and osteoblast differentiation with cells from WT and *Gnas*^m−/+^ are necessary to determine the mechanisms underlying these *in vivo* osteoblast changes.

By contrast, endosteal osteoclast numbers were increased *in vivo* in *Gnas*^+/p−^ but not in *Gnas*^m−/+^ mice when compared to WT. However, *in vitro* differentiation of either *Gnas*^+/p−^ or *Gnas*^m−/+^ bone marrow macrophages to osteoclasts was enhanced. *Gnas* mutant osteoclasts also had increased bone resorption activity *in vitro*, with paternal mutants showing a more dramatic increase than the maternal mutants. These results suggest that haploinsufficiency of Gsα leads to increase in osteoclastogenesis *in vitro*, but additional factors *in vivo* [such as sclerostin which is increased in *Gnas*^+/p−^ cortical bone (Fig. 6c–g) and decreased in *Gnas*^m−/+^ cortical bone (Supplementary Figure 6b)] and imprinting effects may contribute to the persistent cortical bone phenotype and increased endosteal osteoclasts that we observe only in the paternal mutants. Although our *in vitro* experiments suggest a cell-autonomous role for Gsα in osteoclasts, future studies ablating *Gnas* specifically in the osteoclast lineage (using LysozymeM-Cre^42^ or RANK-Cre^10^) would further elucidate the role of *Gnas* in osteoclast differentiation and function as well as its impact on skeletal development and remodeling.

Interactions between the Wnt signaling pathways and cAMP/PKA have been reported previously^43^,^44^. Wnt signaling has been established to inhibit osteoclast differentiation via canonical^10^,^45^ (β-catenin) and non-canonical (cAMP/PKA) pathways^10^. Wnt signaling inhibits osteoclastogenesis by stabilizing β-catenin^10^, a primary component of the canonical pathway, and also by increasing levels of OPG^27^,^45^,^46^. In addition, Wnt also enhances cAMP and PKA phosphorylation, and suppresses Nfatc1 by inhibiting its nuclear translocation and autoamplification of Nfatc1^10^. However, these reports did not directly demonstrate a role for Gsα in osteoclast differentiation. Here, we show that reduced *Gnas*/Gsα increased osteoclast differentiation and resorption function via cAMP/PKA and the Wnt/β-catenin pathway. Our results not only support an important role for Gsα signaling in osteoclastogenesis but, together with previous reports^10^, also suggest crosstalk between Gsα/cAMP/PKA and canonical Wnt/β-catenin signaling pathways to regulate osteoclast differentiation.

We did not detect significant trabecular bone effects in either *Gnas*^+/p−^ or *Gnas*^m−/+^ mice, however our data suggested that trabecular bone may also be effected, although more mildly. The trabecular bone effects in our heterozygous germline deletion of *Gnas* were more subtle compared to the stronger effects in cell-specific *Gnas* null mouse models^6^,^8^,^9^,^29^ and could be due to differences in Gsα levels in cells that interact to regulate bone. Additionally or alternatively, locally-acting factors could differentially affect cortical and trabecular bone. We found that expression of Sost, a Wnt inhibitor that was recently determined to induce osteoclast formation and activity^47^, was increased in the cortical bone of *Gnas*^+/p−^ mice, however was extremely low in trabecular bone and could not be reliably quantified (Supplementary Figure 6a); this finding has been consistently noted in published^48^ and unpublished data (J. Wu *et al*). If Sost levels are indeed much lower in trabecular bone, this could be an explanation for the stronger cortical bone phenotype. Further analyses of these mutants will provide information to delineate the roles of Gsα in cortical and trabecular bone.

Our data suggest that osteoclast cell autonomous effects of *Gnas* mutation together with extrinsic factors that act on osteoclasts influence the observed *in vivo* bone phenotype. This study reveals important roles for *Gnas*/Gsα signaling in bone remodeling through regulation of osteoclastogenesis and osteoclast resorption activity, with potential implications for drug development approaches to treating diseases that affect cortical bone quality.

## Materials and Methods

### Animals

Mice with heterozygous deletion of maternal and paternal alleles of *Gnas* were described previously^20^. To generate mice with paternal inheritance of the deletion (*Gnas*^+/p−^), male mice carrying a heterozygous deletion in exon 1 of *Gnas* (maintained on a SvEv background) were crossed to female CD1 wild-type mice. Mice with maternal inheritance of the *Gnas* deletion (*Gnas*^m−/+^) were generated by crossing female mutant mice to male wild-type mice. In both breeding schemes, *Gnas*^+/+^ littermates were used as wild-type controls in all experiments. Only males were used for all experiments to minimize variability and because males were previously reported to have a more consistent phenotype^20^. All animal experiments were performed in accordance with the relevant regulations and guidelines and were approved by the Institutional Animal Care and Use Committee (IACUC), University of Pennsylvania.

Osx1-GFP::Cre^49^ and Gsα(fl/fl)^50^ mice were described previously. Because these mice have a mixed genetic background (C57BL/6 and CD1), littermates were used as controls. Female conditional heterozygous mice [Osx1-GFP::Cre + ;Gsα^fl/+^] were mated to control males (mixed background) to generate heterozygous mice with disruption of the maternal allele (Osx1-GFP::Cre + ;Gsα^m−/+^ referred to as Osx-Cre;*Gnas*^m−/+^ in this study). Male conditional heterozygous mice were mated to wild-type females to generate heterozygous mice with disruption of the paternal allele (Osx1-GFP::Cre + ;Gsα^+/p−^ referred to as Osx-Cre; *Gnas*^+/p−^ in this study). Control mice with Osx1-GFP::Cre [Osx1-GFP::Cre + ; Gsα^+/+^ referred to as Osx-Cre; *Gnas*^+/+^ in this study] and without [Gsα^fl/+^ and Gsα^+/+^ referred to *Gnas*^*fl/*+^ and *Gnas*^+/+^ in this study] were also analyzed to ensure that the Osx1-GFP::Cre transgene did not confer a phenotype. Genotyping used genomic DNA isolated from tails and previously published protocols^6^,^8^,^51^. These animals were housed in the Center for Comparative Medicine at the Massachusetts General Hospital, and all experiments were approved by the hospital’s Subcommittee on Research Animal Care.

### Microcomputed tomography (μCT)

Femurs from 2 week, 3 month and 9 month old mice were harvested and scanned by μCT (μCT35, SCANCO Medical AG, Brüttisellen, Switzerland). For trabecular bone analysis, scans were performed at the distal femoral metaphysis 0.4 mm proximal to the growth plate. For cortical bone, mid-diaphysis of femurs were scanned. All scans were performed at a resolution of 6 μm per slice using a X-ray energy of 55 kvp and an integration time of 300 ms. A total of 125 slices were analyzed using the instrument’s software.

### Mechanical testing

Femurs from 2 week, 3 month and 9 month old mice were subjected to mechanical testing by three-point bending using a custom-made fixture^52^ on an Instron machine. Load was applied until failure and stiffness and peak load were calculated from the load-displacement data^53^. 2D cortical bone μCT images were incorporated into a custom developed Matlab program to determine the moment of inertia.

### Histology

Limbs were fixed in 4% paraformaldehyde for 24–48 hours, decalcified in 10% EDTA for 7 days, processed, and embedded in paraffin. Sections of 5 μm thickness were cut for staining. TRAP staining used the leukocyte acid phosphatase kit (Sigma 387 A). Multi-nucleated osteoclasts (≥3 nuclei) were counted along the endosteal surface at the femoral diaphysis and at the distal femur proximal to the growth plate. Osteoblasts were identified based on their morphology after H&E staining and counted along the endosteum at the diaphyseal region. To determine mineral apposition rate (MAR), mice were injected with calcein (15 μg/g body weight) on postnatal day 10 and xylenol orange (100 μg/g body weight) on postnatal day 13 and sacrificed 24 hours later. MAR was calculated as the distance between the two labels along the femoral mid-diaphyseal region by the number of days between the injections.

### Immunohistochemistry

Paraffin embedded sections were detected with Sost antibody (anti-goat; R&D systems, AF1589). Antigen retrieval was performed by treating the sections with Proteinase K (20 μg/ml, Roche) at 37 °C for 15–20 minutes. Sections were then treated with 3% hydrogen peroxide for 10 minutes followed by blocking with 5% BSA and 10% donkey serum in 1X PBST. Sections were incubated with the Sost antibody at 1:100 dilution overnight, then donkey anti-goat IgG-HRP linked antibody (Santa Cruz Biotechnology) at 1:1200 for 1 hour and developed using DAB chromogen (Life Technologies). Three to five images were taken along the mid-diaphyses of femur, starting from 2 mm proximal to the growth plate, and Sost positive cells were quantified.

### Real-time PCR

Femur and tibiae from mice were stripped of soft tissue, ends were cut and bone marrow was flushed to obtain only the diaphyseal bone region. Cortical bone pieces were frozen in liquid nitrogen and crushed with stainless steel beads (7 mm diameter, Qiagen) using a tissue lyser (TissueLyser LT, Qiagen). RNA was then extracted with Trizol (Thermo Fisher Scientific) using manufacturer’s instructions. cDNA was prepared using High-Capacity RNA-to-cDNA kit (Thermo Fisher Scientific). Real-time qRT-PCR was performed using SYBR Green method. Primer sequences are as follows: Gsα: 5′-GCGCGAGGCCAACAAAAAGAT and 5′-TGCCAGACTCTCCAGCACCCAG; Sost: 5′-CCAGGGCTTGGAGAGTACC and 5′-GCAGCTGTACTCGGACACATC; Sfrp4: 5′-AGAAGGTCCATACAGTGGGAAG and 5′-GTTACTGCGACTGGTGCGA.

### Osteoclast differentiation and function

Following our standard procedures^54^, bone marrow from 6–8 week old mice was flushed from femurs and tibiae using a 26-G needle into α-MEM (Gibco) with 10% fetal bovine serum and 1X antibiotics. After lysis of RBCs, cells were cultured overnight with recombinant M-CSF (Peprotech, NJ) at 5 ng/ml. Next day, non-adherent cells were harvested and reseeded with M-CSF at 30 ng/ml for 3 additional days to acquire bone marrow macrophages (BMM). After 3 days, BMM were collected and cultured with M-CSF (30 ng/ml) and RANKL (Peprotech, 150 ng/ml) at 5 × 10^5^ cells per well in a 6-well plate for RNA or protein and 5 × 10^3^ cells per well in a 96-well plate for TRAP staining.

For pit formation assays^54^, BMM were first differentiated into osteoclasts with M-CSF (30 ng/ml) and RANKL (150 ng/ml) for 3 days. Osteoclasts were then seeded at 1 × 10^4^ cells on dentine slices for 48 hours and percent resorption area was determined. Mean of resorption area for WT was set to 1.

### Flow cytometry

Bone marrow was flushed from 6–8 week old mice using 1X PBS with 2% FBS. Red blood cells were lysed using 1X RBC lysis buffer (Biolegend). Cells were washed twice in 1X PBS with 0.5% BSA, then blocked using rat IgG (TruStain fcX, Biolegend) for 10 minutes on ice. Cells (1 × 10^6^ cells in 100 μl) were then detected with antibodies at 0.2 μg per million cells for 30 minutes on ice protected from light. The antibodies used for flow cytometry to detect osteoclast precursors were the following: anti-mouse CD3-BV421 (BD Biosciences #564008), anti-mouse CD45R-Alexa Fluor488 (BD Biosciences #557669), anti-mouse CD11b-APC (BD Biosciences #553312), anti-mouse CD115-PE (BD Biosciences #565249). Flow cytometry was conducted with a LSR II (BD Biosciences) and data were analyzed using FlowJo software.

### Western blot

Whole cell extracts were obtained using RIPA buffer (Sigma) while nuclear fraction was isolated using NE-PER nuclear and cytoplasmic extraction kit (ThermoFisher Scientific) along with proteinase (Sigma) and phosphatase inhibitor cocktails (Sigma). Protein samples were separated by 4–10% SDS-PAGE and transferred to nitrocellulose membranes. Blots were blocked with 5% milk in 1 X TBST, then incubated with primary antibodies overnight at 4 °C. Primary antibodies used in this study were pCREB, β-catenin, cyclin D1, GAPDH, Histone H3 (all from Cell Signaling, MA, USA) and Nfatc1 (Santa Cruz Biotechnology, CA, USA); all were used at 1:1000 dilution. Gapdh and Histone H3 were used at 1:5000 dilution. Membranes were washed 3 times in TBST and incubated in secondary antibodies conjugated to HRP at 1:6000 dilution for 1 hour at room temperature. Blots were developed and densitometry was quantified with a LI-COR C-Digit blot scanner.

### cAMP Measurement

Intracellular cAMP levels were measured in macrophages from WT and *Gnas*^+/p−^ mice using a cAMP EIA system (GE Healthcare, Little Chalfont, UK). The assay was performed according to the mannufacturer’s instructions. Data were normalized to total protein and WT was set to 1.

### Statistical analyses

One-way ANOVA with Tukey post-hoc tests was used for comparing WT (*Gnas*^+/+^), *Gnas*^+/p−^ and *Gnas*^m−/+^ groups for μCT and mechanical testing data. WT data from paternal and maternal litters were pooled. Two-way ANOVA with Bonferroni post-hoc tests was used for analyzing osteoclast differentiation assays. Student’s t-test or one-way ANOVA was performed to compare real time qRT-PCR, pit formation data and sclerostin immunohistochemistry data after setting littermate controls to 1 when comparing two or more than two groups respectively. p < 0.05 was considered statistically significant.

## Additional Information

**How to cite this article:** Ramaswamy, G. *et al*. Gsα Controls Cortical Bone Quality by Regulating Osteoclast Differentiation via cAMP/PKA and β-Catenin Pathways. *Sci. Rep.* **7**, 45140; doi: 10.1038/srep45140 (2017).

**Publisher's note:** Springer Nature remains neutral with regard to jurisdictional claims in published maps and institutional affiliations.

## Supplementary Material

Supplementary Information
