# Supplementary material for: Gsα Controls Cortical Bone Quality by Regulating Osteoclast Differentiation via cAMP/PKA and β-Catenin Pathways
Source: Sci Rep. 2017 Mar 24;7:45140. doi: 10.1038/srep45140 (PMC5364530; doi:10.1038/srep45140)
Supplement: Supplementary Information [file srep45140-s1.pdf]

Gsα Controls Cortical Bone Quality by Regulating Osteoclast Differentiation via cAMP/PKA and β-Catenin Pathways

Girish Ramaswamy[1,5] Hyunsoo Kim[4] Deyu Zhang[1,5], Vitali Lounev[1,5], Joy Y Wu[6], Yongwon Choi[4], Frederick S Kaplan[1,2,5], Robert J. Pignolo[7], Eileen M. Shore[1,3,5]

Supplementary Table 1 Bone phenotype in *Gnas* mutant mice at 2 week, 3 and 9 month mice.

	2 weeks			3 mon			9 mon		
	WT	*Gnas*$^{+/p-}$	*Gnas*$^{m-/+}$	WT	*Gnas*$^{+/p-}$	*Gnas*$^{m-/+}$	WT	*Gnas*$^{+/p-}$	*Gnas*$^{m-/+}$
Size and Geometric Analyses									
Body Weight (g)	8.82 ± 0.9	6.88 ± 0.35**	7.86 ± 0.64*	40. 9 ± 3.4	35.97 ± 3.56*	46.6 ± 0.67	53.28 ± 7.35	48.41 ± 4.24	59.91 ± 4.37
Femur Length (mm)	9.68 ± 0.47	8.8 ± 0.37*	8.57 ± 0.56*	17.19 ± 0.26	16.29 ± 0.5**	17.04 ± 0.02	17.02 ± 0.45	16.29 ± 0.18**	16.92 ± 0.72
Moment of Inertia (mm^4)	0.03 ± 0.002	0.02 ± 0.002**	0.02 ± 0.0.001**	0.23 ± 0.05	0.128 ± 0.06**	0.29 ± 0.02*	0.31 ± 0.08	0.25 ± 0.06**	0.36 ± 0.07
TV - Cortical (mm^3)	0.75 ± 0.1	0.66 ± 0.03*	0.72 ± 0.13*	1.71 ± 0.17	1.41 ± 0.21**	1.87 ± 0.04*	1.88 ± 0.25	1.82 ± 0.17	2.08 ± 0.2
Cortical Porosity	10.26 ± 0.86	11.01 ± 1.32	17.06 ± 2.33**	4.74 ± 0.36	5.41 ± 0.64	4.5 ± 0.18	5.51 ± 1.12	6.02 ± 0.8	5.17 ± 0.81
Trabecular Bone Analyses									
TV (mm^3)	0.531 ± 0.025	0.416 ± 0.045	0.436 ± 0.054	2.214 ± 0.254	2.004 ± 0.352	2.607 ± 0.129	2.73 ± 0.281	3.068 ± 0.317	3.12 ± 0.427
Tb. N (1/mm)	7.697 ± 0.686	7.983 ± 0.18	7.246 ± 0.29	5.746 ± 0.336	5.549 ± 0.515	5.529 ± 0.305	3.81 ± 0.356	3.60 ± 0.455	3.57 ± 0.277
Tb. Th. (mm)	0.029 ± 0.002	0.027 ± 0.001	0.027 ± 0.003	0.043 ± 0.005	0.037 ± 0.007	0.048 ± 0.005	0.048 ± 0.005	0.045 ± 0.003	0.048 ± 0.004
Tb. Sp. (mm)	0.131 ± 0.011	0.126 ± 0.003	0.137 ± 0.007	0.166 ± 0.012	0.175 ± 0.02	0.172 ± 0.011	0.26 ± 0.027	0.278 ± 0.039	0.28 ± 0.025

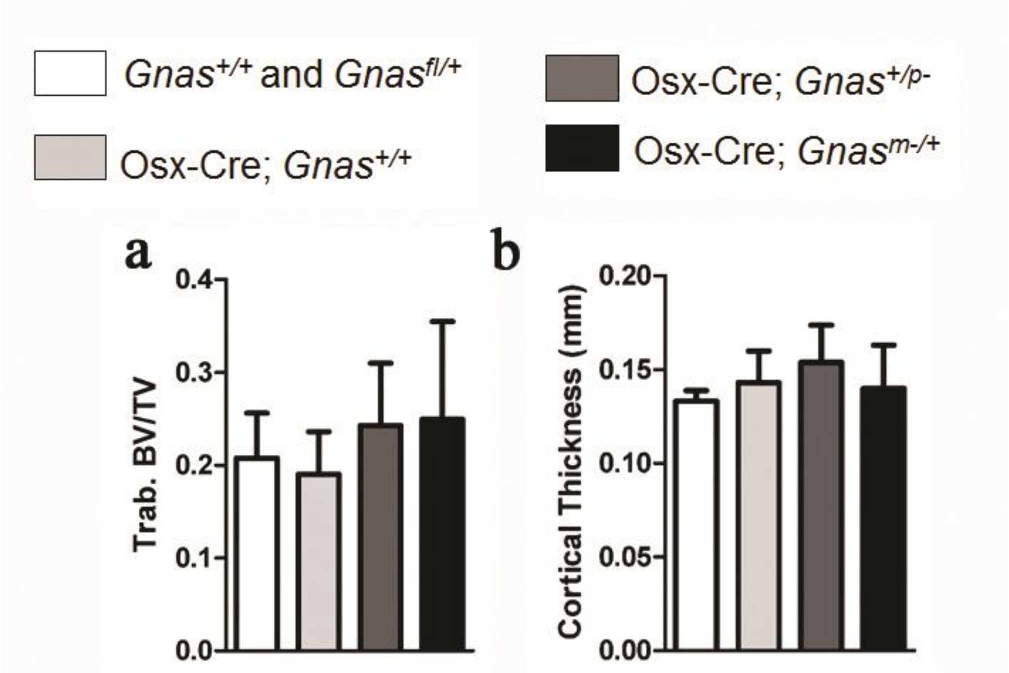

Gnas+/+ and Gnasfl/+
Osx-Cre; Gnas+/+
Osx-Cre; Gnas+/p-
Osx-Cre; Gnasm-/+
a
Trab. BV/TV
0.0
0.1
0.2
0.3
0.4
b
Cortical Thickness (mm)
0.00
0.05
0.10
0.15
0.20

Supplementary Figure 1 Bone phenotype in mice with heterozygous deletion of *Gnas* in osteoblasts. Quantification of (a) trabecular BV/TV and (b) cortical thickness from mice with paternal and maternal inheritance of heterozygous deletion of Gsα in cells expressing osterix at 6 weeks of age (Osx-Cre; $Gnas^{+/p-}$ and Osx-Cre; $Gnas^{m-/+}$) shows no differences compared to age-matched controls ($Gnas^{+/+}$, $Gnas^{fl/+}$ and Osx-Cre; $Gnas^{+/+}$). Data represent mean ± SD. N = 4-7 animals per group.

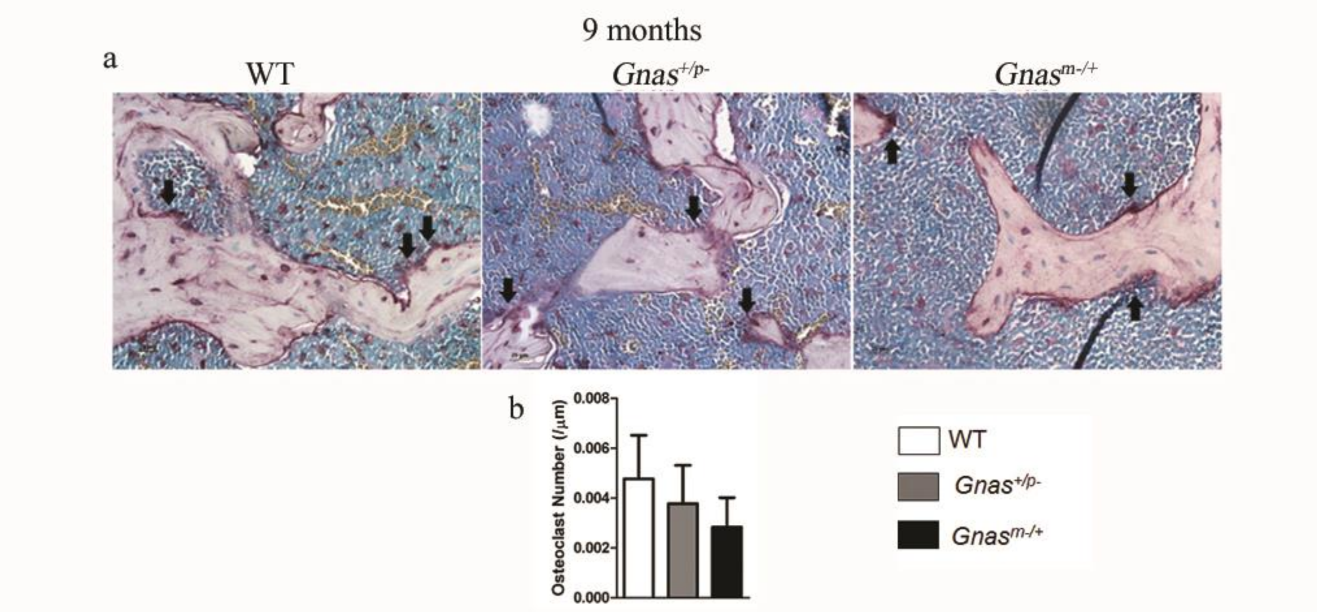
9 months
a
WT
Gnas+/p-
Gnasm-/+
b
Osteoclast Number (/μm)
0.008
0.006
0.004
0.002
0.000
WT
Gnas+/p-
Gnasm-/+

Supplementary Figure 2 No difference in osteoclast number among the genotypes in trabecular bone at 9 months of age. (a) TRAP staining of metaphyseal trabecular bone at the distal femur. (b) Quantification of the osteoclast number per perimeter in trabecular bone in all genotypes. Data represent mean ± SD. N = 3-4 animals per group.

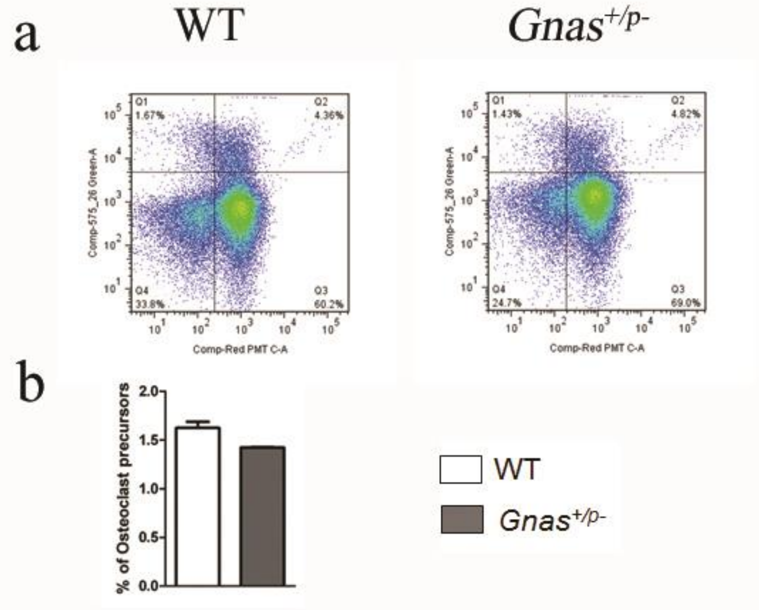
a
WT
Gnas+/p-
Q1
1.67%
Q2
4.36%
Q4
33.8%
Q3
60.2%
Comp-575_26 Green-A
Comp-Red PMT C-A
Q1
1.43%
Q2
4.82%
Q4
24.7%
Q3
69.0%
b
% of Osteoclast precursors
2.0
1.5
1.0
0.5
0.0
WT
Gnas+/p-

Supplementary Figure 3 *Gnas*$^{+/p-}$ mice do not show differences in osteoclast precursor population in the bone marrow. (a) Cells from WT and *Gnas*$^{+/p-}$ bone marrow were analyzed by flow cytometry for CD3^{-} CD45R^{-} CD11b$^{-/low}$ CD115^{high} population and their percent was (b) quantified. Data represent mean ± SD. N = 3 mice per group.

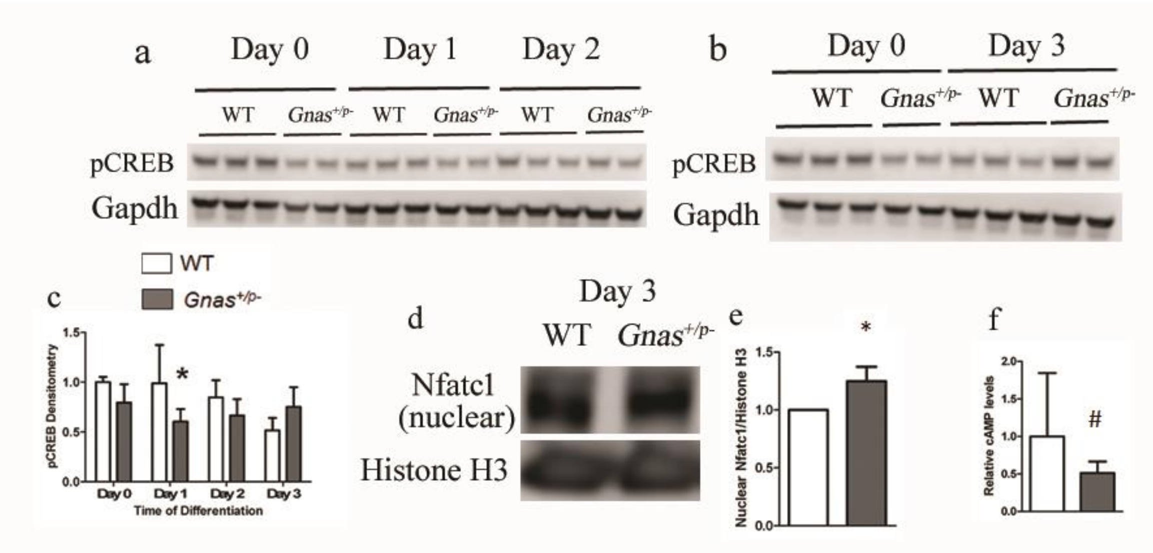

a
Day 0
Day 1
Day 2
WT
Gnas+/p-
pCREB
Gapdh
b
Day 0
Day 3
c
WT
Gnas+/p-
pCREB Densitometry
Time of Differentiation
Day 0
Day 1
Day 2
Day 3
d
Day 3
Nfatc1
(nuclear)
Histone H3
e
Nuclear Nfatc1/Histone H3
f
Relative cAMP levels
#

Supplementary Figure 4 Protein levels of pCREB and Nfatc1 during osteoclast differentiation. Western blots show significant decrease in (a-c) pCREB at day 1 and increase in (d) Nfatc1 at day 3 during osteoclast differentiation in cells from *Gnas*$^{+/p-}$ over WT cells. (d and e) Nfatc1 was increased in nuclear fractions of *Gnas*$^{+/p-}$ over WT cells at day 3 of osteoclast differentiation. (f) cAMP levels were reduced in *Gnas*$^{+/p-}$ macrophages compared to WT cells. WT was set to 1 in e and f. Data represent mean ± SD. Experiments a-c were performed at least 3 times with n = 2-3 animals per group per experiment. Total of 5-7 animals per group from 3 experiments used for quantification. For Nfatc1 nuclear fraction and cAMP measurements, n = 3 biological replicates. *$p < 0.05$, #$p \leq 0.1$.

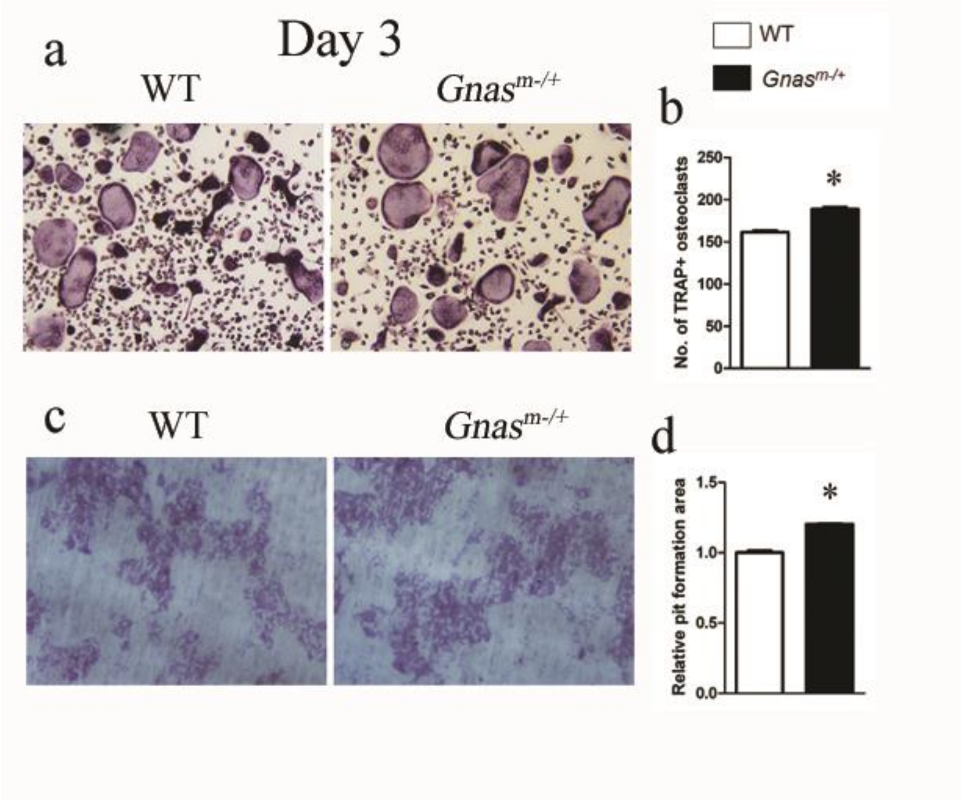

a
Day 3
WT
Gnasm-/+
b
No. of TRAP+ osteoclasts
0
50
100
150
200
250
*
WT
Gnasm-/+
c
WT
Gnasm-/+
d
Relative pit formation area
0.0
0.5
1.0
1.5
*

Supplementary Figure 5. *Gnas*$^{m-/+}$ show marginal increase in osteoclastogenesis. (a, b) Bone marrow macrophages from *Gnas*$^{m-/+}$mice showed marginal increase in osteoclast differentiation. (c, d) Osteoclasts from *Gnas*$^{m-/+}$mice showed modest elevation in resorption areas measured using pit formation assay. WT mean resorption area was set to 1. Data represent mean ± SD. Experiments were performed 2 times with n = 2-3 animals per group per experiment. Total of 4-5 animals per group from 2 experiments used for quantification. *$p < 0.05$.

a

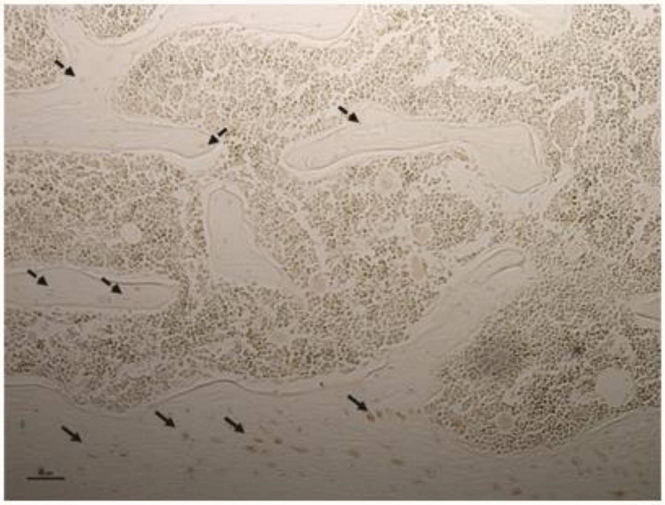

b

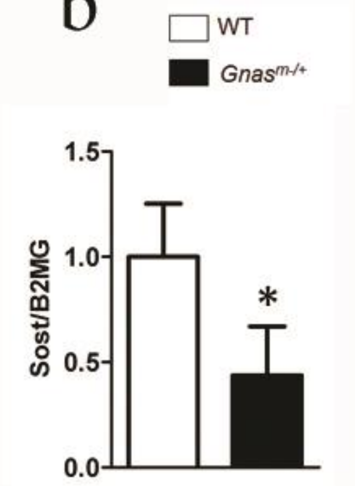

Supplementary Figure 6. Sost expression in trabecular bone and *Gnas*$^{m-/+}$ femurs. (a) Intensity of sclerostin expression was very low and undetectable in trabecular bone as compared to much stronger staining in cortical bone. Solid arrows represent osteocytes staining for sclerostin in cortical bone and dotted arrows show osteocytes in trabecular bone that does not show detectable levels of sclerostin expression. (b) Sost mRNA expression was significantly reduced in cortical bone of 9 months old *Gnas*$^{m-/+}$ mice as compared to WT littermates. Mean of WT was set to 1. Data represent mean ± SD. Total of 4-5 animals per group used for quantification. *$p < 0.05$.
